# Phage Therapy in Bacterial Infections Treatment: One Hundred Years After the Discovery of Bacteriophages

**DOI:** 10.1007/s00284-016-1166-x

**Published:** 2016-11-28

**Authors:** Agata Anna Cisek, Iwona Dąbrowska, Karolina Paulina Gregorczyk, Zbigniew Wyżewski

**Affiliations:** 1 Division of Microbiology, Department of Preclinical Sciences, Faculty of Veterinary Medicine, Warsaw University of Life Sciences-SGGW, Ciszewskiego 8, 02-786 Warsaw, Poland; 2 Division of Immunology, Department of Preclinical Sciences, Faculty of Veterinary Medicine, Warsaw University of Life Sciences-SGGW, Ciszewskiego 8, 02-786 Warsaw, Poland

## Abstract

The therapeutic use of bacteriophages has seen a renewal of interest blossom in the last few years. This reversion is due to increased difficulties in the treatment of antibiotic-resistant strains of bacteria. Bacterial resistance to antibiotics, a serious problem in contemporary medicine, does not implicate resistance to phage lysis mechanisms. Lytic bacteriophages are able to kill antibiotic-resistant bacteria at the end of the phage infection cycle. Thus, the development of phage therapy is potentially a way to improve the treatment of bacterial infections. However, there are antibacterial phage therapy difficulties specified by broadening the knowledge of the phage nature and influence on the host. It has been shown during experiments that both innate and adaptive immunity are involved in the clearance of phages from the body. Immunological reactions against phages are related to the route of administration and may vary depending on the type of bacterial viruses. For that reason, it is very important to test the immunological response of every single phage, particularly if intravenous therapy is being considered. The lack of these data in previous years was one of the reasons for phage therapy abandonment despite its century-long study. Promising results of recent research led us to look forward to a phage therapy that can be applied on a larger scale and subsequently put it into practice.

## History of Bacteriophage

The history of phage therapy could be separated into four timespans, according to Summers et al. [48].

### Early Enthusiasm

Bacteriophages were discovered by English microbiologist Twort in 1915 [1] but “the bacteriophage phenomenon” era began after publication in 1917 by a French-Canadian microbiologist Felix d’Herelle. During his investigations, he observed “invisible microbes” in filtrates of stool from patients suffering from dysentery that were “antagonistic” to bacteria. He surmised that this filterable virus, “ultraviruses,” was a cofactor of bacterial infection. However, he proved that phage titers increased in disease progression and peaked during recovery. After those successes, d’Herelle branched out his investigations on humans. At first, he had tested the safety of phage suspension on himself, his co-workers, and family, then on patients suffering from “bacillary dysentery” and cholera (since 1919). After that, phages were applied as a therapy to wound recovery. Another experiment that focused on the healing value of phages investigated *Salmonella gallinarum* as an infectious agent of avian typhosis (published in 1926). This test also confirmed phage protection, as well as it did against other species, like *Pasteurella multocida* (bovine hemorrhagic septicaemia, published in the same year). Nevertheless, the first publication about phage therapy described the work of Bruynoghe and Maisin. Their results were published in 1921 [1, 48].

### Critical Scepticism

Despite the fact that many early experiments of phage therapy reported positive results, there were also some disappointments. In 1934, a report was published in which previously released data were criticized. In the author’s opinion, the biological viral nature of phage was not known well enough, both their strengths and limitations. Moreover, the report showed mistakes such as no standard in phage preparation and no criteria to compare the investigations’ results [1].

### Abandonment

War World II and the discovery of antibiotics reversed away the study in bacteriophage investigation, especially in the USA. The effortlessness of production, broad spectrum of activity, and stability in the preparation process were the advantages of antibiotics. In Europe, by contrast, two military-expanded countries (the Soviet Union and Germany) used phages as a medical treatment for healing wounds. In the Soviet Union, applications of phages were mainly driven by cost-effective and ideational motives (“preponderance” of Soviet science over the capitalist West) [47]. Moreover, The State Serum and Vaccine Institute in Tbilisi, Georgia, founded, among others, by d’Herelle was one of the major centres of phage therapy of that time [49]. A curious about phage therapy is the fact that the Pasteur Institute, the mother institution of d’Herelle where he worked on bacteriophages, obtained their bacterial viruses mainly from Russia or Georgia, even at the present time [1].

Another reason of abandoning the bacteriophage therapy in post-war time was the problem of phage-resistant bacteria, an unwittingness of pathogenic mechanisms in bacteria and of the nature of interactions between phage and their host. This lack of knowledge also included DNA restriction, an absence of models of animal diseases and listed lapses in experimental design [2].

### Recent Interest and Reappraisal

In the 1970s, in Pakistan several experiments of the use of bacteriophages (prepared in the USSR), in the treatment of cholera were sponsored by WHO [27, 29]. A conclusion of these articles could be a statement that treatment of cholera with bacteriophage is not as effective as therapy with antibiotic (tetracycline); however, anti-cholera phage can selectively reduce the majority of vibrios without interfering with other intestinal microorganisms, and without any noticeable toxic effect on the patient. Therefore, bacteriophage might be a helpful study instrument.

Another set of articles about phage therapy applied as the treatment of diarrohea (models of mice and farm animals infected with *Escherichia coli*) concluded that bacteriophages could be employed both in treatment and prophylaxis [41–44]. These works were the beginning of western phage renaissance, which was propelled also by the rich trove of Soviet and Polish work. Research in Poland has been done mainly in connection with the Hirszfeld Institute of Immunology and Experimental Therapy in Wroclaw and has involved thousands of patients. Furthermore, those studies are deemed to be one of the most precisely documented [1]. In the whole history of bacteriophage therapy, there were a lot of investigations including also other etiological factors of diseases of both animals and humans such as *Acinetobacter*, *Burkholderia*, *Citrobacter*, *Enterobacter*, *Enterococcus*, *Proteus*, *Pseudomonas*, *Shigella*, *Staphylococcus* and *Streptococcus* [47, 48]. The newest scientific research in the field of phage therapy was presented in the following sections of this article.

## How Do Bacteriophages Kill Bacteria?

The increasing number of antibiotic-resistant bacterial strains is a serious problem in contemporary medicine. What is important is the fact that these bacteria do not implicate resistance to phage lysis mechanisms. Lytic bacteriophages are able to kill antibiotic-resistant bacteria at the end of the phage infection cycle. Most of them utilize two-component lysis systems to destroy a bacterial cell wall in order to release progeny virions. Thus, the development of phage therapy is a potential way to improve treatment of bacterial infections.

The course of a replication cycle is the criterion to divide bacteriophages into lytic and lysogenic ones. The release of lytic phage progeny from infected cells requires bacterial lysis. Scientific studies on phage lytic mechanisms contribute to the development of phage therapy. Some lytic bacteriophages use single proteins, amurins, to inhibit the synthesis of peptidoglycan [56]. However, most of them utilize two groups of proteins to kill the host cell. The first ones, holins, synergize with the second ones, endolysins, to cause lysis. Together, they make up the holin–lysin systems [10, 23].

Holins are involved in the host cell lysis triggering process. Their role is to perforate the host cytoplasmic membrane and thus to cooperate with endolysins by giving them an access to bacterial peptidoglycan. Therefore, holins determine the time of bacterial lysis. Acting at a precise time point, they control bacterial murein accessibility for phage endolysins and thus they synchronize the activity of the holin–lysin system with the late-phase events of the phage replication cycle [8, 39].

The primary structure of holins is not well conserved in evolution. However, differences in the amino acid sequences among holins are not reflected by their function. Every holin has at least one hydrophobic transmembrane domain (TMD) as well as C-terminal hydrophilic domain that carries a high electric charge. There are three classes of holins. Class I includes proteins that have more than 95 amino acid residues in length. These holins has three TMDs. Holins belonging to Class I are represented by *Staphylococcus aureus* bacteriophage p68 hol15 protein and *Escherichia coli* phage λ S105 protein. Holins belonging to class II have 65–95 amino acid residues in length and they have two TMDs. Lambdoid phage 21 S protein and *Clostridium perfringens* bacteriophage Ф3626 hol3626 protein are members of this class. Class III holins form only one TMD and are represented by the phage ФCP26F holin [8, 31, 38, 39, 50].

S105, the product of the phage λ S gene expression, is the most widely studied holin. S105 localizes the plasma membrane and, at a proper time point, it forms lethal lesions (holes) in lipid bilayer [8]. The average diameter of membrane holes is more than 340 nm [51].

Phage endolysins are enzymatic proteins responsible for cell wall degradation [54]. Bacteriophages use them to hydrolyze the peptidoglycan of the infected bacteria [37]. Endolysins perform activities of endopeptidase, amidase, glycosidase or lytic transglycosylase to kill bacterial cell by murein destruction [9, 22, 30]. Acting at the end of the phage replication cycle, endolysins promote the release of progeny virions. Endolysins directed against gram-negative bacteria have different structures from those targeting gram-positive ones, reflecting differences between enzyme targets. Gram-negative bacteria are surrounded by the outer membrane, and therefore the access to the cell wall is restricted from the outside. It seems to be the reason why endolysins targeting gram-negative bacteria are small globular proteins composed of only one domain, called enzymatically active domain (EAD) [37], whereas endolysins directed against gram-positive microorganisms have also cell wall binding domain (CBD) [4, 23, 24]. Enzyme targeting gram-positive bacteria binds to the cell wall through its CBD and thus remains immobilized on peptidoglycan surface. CBD contributes to the hydrolytic effects of endolysin by synergizing with EAD that performs an acatalytic function of enzymatic protein. During this process, endolysin remains tightly bound to one site of the peptidoglycan structure [37].

The holin–lysin system is responsible for termination of the phage infection cycle at a specific time point. The effect of endolysins on bacterial cell wall is subjected to precise timing through mechanism explained by the dual-start model. In this model, the time of bacterial lysis is dependent on the proportion between holin and its antagonist, antiholin. Both of them are encoded by an open reading frame. The ratio of holin to antiholin is strictly regulated by controlling their expression at the translational level. Elevation of the holin–antiholin ratio is followed by the loss of plasma membrane integrity [39], which allows endolysin to reach periplasm and begin to degrade the host peptidoglycan [51].

## Interactions Between Bacteriophages and Host Immune System During Phage Therapy

Phage therapy may carry a risk of immunological reactions, that is why studies about interactions between phages and immunity are very important for the rational use of this treatment. Immune response against bacteriophages depends on the localization of bacterial infection and the injection site of therapeutic phages. Under physiological conditions, some phages are associated with the eukaryotic component of a gut microbiota and ingested food [35]. High frequency of natural contact of animals/humans with various types of phages is evidenced by the anti-phage antibodies detected in the sera of different species (e.g. human) [44]. Furthermore, an oral administration of phages during phage therapy of bacterial infection caused by *Staphylococcus*, *Klebsiella*, *Escherichia*, *Proteus* and *Pseudomonas* induces the production of antibodies as well [7]. There is no evidence of immunological complications after the consumption of large amounts of phages [36]. Moreover, the topical applications of phages have not shown any side effects [57]. A different situation is observed in the other internal organs and blood stream, which are not natural environment for phages. Intravenous administration of bacterial pathogens strongly stimulates both innate and adaptive immunity [28]. Furthermore, studies show that phages can penetrate into the circulation, regardless of the route of administration [7]. If there are no host bacteria for specific phages, they are rapidly removed from the blood and internal organs by phagocytic cells [28]. Moreover, bacterial predators are internalized and eliminated by cells of the reticuloendothelial system of liver and spleen. Interestingly, Kupffer cells (specialized macrophages which are located in the liver) can phagocyte phages four times faster than spleen macrophages, which suggests that arrested bacterial pathogens in spleen may stimulate lymphocytes to produce antibodies [7, 14]. Innate immunity, known as the first line of defence, is often sufficient to eliminate pathogens before the activation of adaptive immune response. Studies have shown that patients subjected to phage therapy were characterized by the decreasing number of mature neutrophils and the increasing number of neutrophil precursors in the peripheral blood [55]. These results indicated that phage preparations can activate innate immune response, which is helpful in the clearance of bacterial infection. On the other hand, phages can affect immune cells’ metabolic activities as well. For instance, studies have shown that bacteriophages markedly inhibit ROS production in response to pathogenic bacteria and suggest that phages decrease antibacterial innate immunity [32]. However, the relevance of these findings in relation to clinical situations is discussed.

During phage therapy, phages are able to induce specific antibodies (neutralizing antibodies) against them, which usually inhibit phage effectiveness to lyse the targeted bacteria in vivo [15, 16, 25, 46]. In fact, neutralizing antibodies are defined as antibodies that bind epitopes within those parts of the virion essential for infecting the host cells [13]. However, it is not clear how long this type of antibodies will remain in circulation. Concentration of neutralizing antibodies depends on many aspects, for instance, (a) the route of phages administration (topical and oral administration cause a small increase of antibodies) and (b) the dosage protocol [47]. Studies have shown that anti-phage neutralizing antibodies are probably one of the most important factors responsible for the efficacy limitation of phage therapy [44]. However, Sulakvelidze et al. [46] suggested that the development of neutralizing antibodies should not be a significant problem during the initial treatment of acute infections, because the kinetics of phage action are much faster than the host’s production of neutralizing antibodies. Nevertheless, anti-phage antibodies can be an obstacle if they are still present at the time the second course of treatment is administered. There are three ways to solve that problem. It might be envisaged to (a) repeat phage administration, (b) increase the phage concentration or (c) use different phages because resistance is different from one phage to another [46]. Despite the fact that anti-phage neutralizing antibodies occur during phage therapy, there is also an increasing level of non-neutralizing antibodies, IgM and later IgG, and enhancement of immune response after subsequent injections of some types of phages [3, 5].

Apart from the humoral immune response, cellular immunity also plays an important role against phages. Langbeheim has shown that subcutaneous injection of MS-2 phages resulted in a strong hypersensitivity reaction in guinea pigs. Similar results have been obtained in vitro [21]. However, some other studies indicated that cellular immune responses play only a slight role in phage inactivation. They showed that the clearance of T7 phage in T cell-deficient mice was similar to that observed in the wild-type mice [45]. In view of the contradictory results, this issue requires further study. Interestingly, some studies have shown that phages can exert immunosuppressive activity. Over the study upon the role of bacteriophages in the development of transplantation tolerance, Górski observed that phages can inhibit the activation of T cells [17]. Moreover, Kniotek indicated that after phage administration the humoral immunity is decreased as well [20].

Altogether results suggest that it is very important to test the immunological response of every single phage, particularly if intravenous therapy is being considered. However, previous clinical and animal trials have not resulted in serious immunologic reactions during phage therapy [28].

## Phage Therapy Now

Bacteriophages have been studied and used to control bacterial infections of patients in Poland, Georgia and Russia for nearly a century. Recently, more countries, including France, Belgium, Switzerland (Phagoburn project) and the USA, decided to join [33]. Phage Therapy Center of the Hirszfeld Institute of Immunology and Experimental Therapy in Wroclaw offers its patients experimental therapies against several bacterial diseases, including those caused by *Acinetobacter*, *Burkholderia*, *Citrobacter*, *Enterobacter*, *Enterococcus*, *Escherichia*, *Klebsiella*, *Morganella*, *Proteus*, *Pseudomonas*, *Shigella*, *Salmonella*, *Serratia*, *Staphylococcus* and *Stenotrophomonas*. Based on the data published by the Centre, 35–50% of patients treated there got positive therapy results. So far, the Centre does not offer treatment for infections caused by *Streptococcus spp.*, *Mycobacterium tuberculosis*, *Propionibacterium acnes*, *Borrelia* spp., *Helicobacter pylori*, and *Haemophilus influenza*, nor *Chlamydia* spp. [19]. As for Eliava Phage Therapy Center in Tbilisi, phages are used there for the treatment of infections caused by *Enterococcus faecalis* of different serovars, *E. coli* (O11, O18, O20, O25, O26, O44, O55, O113, O125, O128), *Proteus vulgaris*, *P. mirabilis*, *Pseudomonas aeruginosa*, *Salmonella* (Paratyphi A, Paratyphi B, Typhimurium, Enteritidis, Heidelberg, Newport, Cholerae Suis, Oranienburg, Dublin, Anatum), *Shigella flexneri* (serovars 1, 2, 3, 4), *Sh. sonnei* (serovar 6), *Sh. newcastle*, *Staphylococcus aureus*, *S. epidermidis*, *S. saprophyticus*, and *Streptococcus pyogenes*, *S. sanguis*, *S. salivarius* and more [12]. In order to increase the antimicrobial activity and to decrease the risk of the phage resistance development, each treatment is a cocktail that contains at least a few bacteriophage strains. Therapy may be executed with the use of lytic or lysogenic phages. In case of the former, even though it is a method of choice, there is still a concern that phage therapy may worsen the health condition of patients since viral lytic cycle is followed by a release of bacterial endotoxins. In contrast, the use of temperate (lysogenic) phages leaves the possibility of transfer of virulence genes into so far non-virulent microbionts of patients. An extensive review over recent bacteriophage experimental trials has been described elsewhere [53].

Evaluation of phage potential in the treatment of human infections is an ongoing process. In the year 2016 alone, a few interesting papers of in vivo experiments on bacteriophages have been published. In the first one, a hypervariable region of a cell wall protein named PIPEF (phage infection protein from *Enterococcus faecalis*) was identified as a crucial factor of phage tropism for *E. faecalis* strains [11]. In vivo testing of phage predation on a gnotobiotic mouse model showed that through mutations in this hypervariable region of PIPEF *E. faecalis* acquired phage resistance.

Simultaneously to the mechanisms of acquired phage resistance in bacteria, possible ways of phage delivery to specific destinations are being studied. In Singla et al. [40], different approaches of incorporation of *Klebsiella pneumoniae* phage particles into liposomes as well as biodistribution of liposome-entrapped phages in various organs of BALB/c mice were evaluated. It turned out that liposome-entrapped phages were a few times more stable in blood and organs than free phages. These results allowed to assume that because of longer bioretentivity rates of those phages in lungs and kidneys, it makes them a suitable candidate for the treatment of *K. pneumoniae*-associated respiratory tract and urinary tract infections.

Interestingly, phage therapy may be used in a far different manner. Prophylactic use of bacteriophages resembles that described for bacterial probiotics. In essence, phages administered orally can eliminate diarrheic pathogens like *Salmonella* spp., *Clostridium difficile* and *E. coli*. They can also—if designed to do so—modulate the gastrointestinal microbiota composition in a preferred way, bringing further benefits for the host [1].

Also, phages were used to control the spread of *Campylobacter jejuni* and *C. coli* infections in chickens [6]. Although *C. jejuni* does not significantly influence the wellbeing of chickens, it is a human pathogen that does not only induce the food poisoning, but is also responsible for its long-term consequences, i.e. development of Guillain–Barre syndrome, reactive arthritis and post-infectious irritable bowel syndrome [52]. Therefore, decreasing the load of *C. jejuni* in poultry products decreases the risk of people getting sick, and in that sense phage therapy may also be considered to have an indirect “probiotic” activity. On a note side, research on the use of phages in other applications, such as construction of antivenoms (phage display), and biocontrol in food manufacturing is still ongoing, yet looks very promising [18, 26, 34].
